# Aphid-induced Defences in Chilli Affect Preferences of the Whitefly, *Bemisia tabaci* (Hemiptera: Aleyrodidae)

**DOI:** 10.1038/srep13697

**Published:** 2015-09-03

**Authors:** Khalid A. Saad, M. N. Mohamad Roff, Rebecca H. Hallett, A. B. Idris

**Affiliations:** 1 School of Environmental and Natural Resource Sciences, Faculty of Science and Technology, National University Malaysia, 43600 UKM, Bangi, Selangor, Malaysia; 2 Horticulture Research Centre, Headquarters, MARDI-UPM, 43400, Serdang Selangor, Malaysia; 3 School of Environmental Sciences, Ontario Agricultural College, University of Guelph, 50 Stone Rd. E., Guelph, Ontario, N1G 2W1, Canada

## Abstract

The sweetpotato whitefly (WF), *Bemisia tabaci*, is a major pest that damages a wide range of vegetable crops in Malaysia. WF infestation is influenced by a variety of factors, including previous infestation of the host plant by other insect pests. This study investigated the effects of previous infestation of host chilli plants by the green peach aphid (*Myzus persicae*) on the olfactory behavioural response of *B. tabaci*, using free-choice bioassay with a Y-tube olfactometer. We analysed volatile organic compounds (VOCs) emitted by non-infested and *M. persicae*-infested chilli plants using solid-phase microextraction and gas chromatography–mass spectrometry. Our results showed that female WFs preferred non-infested to pre-infested plants. Collection and analysis of volatile compounds emitted by infested plants confirmed that there were significant increases in the production of monoterpenes (cymene; 1,8-cineole), sesquiterpenes (β–cadinene, α-copaene), and methyl salicylate (MeSA) compared to non-infested plants. Our results suggest that host plant infestation by aphids may induce production of secondary metabolites that deter *B. tabaci* from settling on its host plants. These results provide important information for understanding WF host selection and dispersal among crops, and also for manipulating WF behaviour to improve IPM in chilli.

The whitefly (WF) *Bemisia tabaci* (Gennadius) (Hemiptera: Aleyrodidae) is an economically important pest in agricultural ecosystems. *Bemisia tabaci* damages a wide variety of crops by feeding on cell sap and by transmitting viral diseases^1^. The first record of *B. tabaci* in Malaysia was in 1935^2^, where it was reported on chilli (*Capsicum annuum* L.), soybean (*Glycine max* (L.) Merr.), and okra (*Abelmoschus esculentus* (L.) Moench) in lowland areas. Although it was initially an unimportant agricultural pest, WF has recently become destructive to many host plants, including brinjal (*Solanum melongena* L.), tomato (*Solanum lycopersicum* L.), and chilli^3^. Feeding by adults and nymphs causes chlorotic leaf spots, leaf fall, and reduced plant growth. Honeydew produced by feeding nymphs coats leaf surfaces, and can result in reduced photosynthetic potential when colonized by moulds. Under heavy infestation, plant height, number of internodes, and quality and quantity of yield can be adversely affected^4^. Crop losses of up to 50% have been attributed to this pest in Malaysia^3^.

Damage caused by *B. tabaci* is compounded by its ability to transmit more than 100 viruses, 90% of which belong to the genus *Begomovirus*^5^. Insecticides have been used extensively to eliminate WF infestations^3^. However, *B. tabaci* has become resistant to a number of pesticide active ingredients^6^ and the chemicals have adverse effects on the natural enemies that control WF populations. For example, avermectin insecticides efficiently control the proliferation of WF larvae on brinjal and tomato, but are toxic to the predator *Macrolophus caliginosus* Wagner (Heteroptera: Miridae), which feeds on WFs^7^.

WF infestation occurs on several host plants in Malaysia and severe infestations of *B. tabaci* on plants offer opportunities to study interspecific interactions with other herbivores^8^. Numerous studies on competition have concluded that previous feeding by one species induces nutritional and allelochemical changes in the host plant that adversely affect the performance of other species that subsequently feed on the same host^9^,^10^,^11^,^12^. Interspecific competition between *B. tabaci* and other herbivores has been investigated by Inbar *et al*. (1999), who reported that first-instar cabbage loopers, *Trichoplusia ni* (Hubner), switched to the WF-free sides of collard leaves that were previously infested with *B. tabaci*. Negative effects on host preference and performance of *Liriomyza trifolii* Burgess and *Liriomyza sativae* Blanchard were observed in the presence of *B. tabaci* on tomato, pumpkin (*Cucurbita pepo* L.), and cucumber (*Cucumis sativus* L.)^13^,^14^.

There is abundant evidence that heavy feeding by sap-consuming insects induces long-term reductions in plant quality^15^,^16^ that could diminish the performance of later-colonizing species^17^. For example, aphid feeding on wheat seedlings induced chemical changes in the plants that subsequently repelled other aphid species^18^. However, reciprocal effects of aphid infestation on WF preferences have not been extensively studied. As such this work aimed to investigate the effects of chilli defence responses induced by pre-infestation with aphids on *B. tabaci* host selection, and to explore the fundamental mechanisms underlying the interspecific interactions between *B. tabaci*, aphids and the host plant. This knowledge will be useful for improving plant protection programs and to increase understanding of induced defenses in plants and their effects on other organisms.

## Results

### Olfactory Bioassay

In olfactory response bioassays, female *B. tabaci* were found to significantly prefer VOCs from non-infested chilli plants over VOCs from pre-infested plants (*t* = 2.31, *P* < 0.05) (Table 1, Exp. 1). Similarly, when WF females were given a choice between VOCs from pre-infested chilli plants and clean air, they showed a significant preference for clean air (*t* = 4.43, *P* < 0.05) (Exp. 2). However, no significant difference in preference was observed between non-infested chilli plants and clean air (*t* = 2.00, P > 0.05) (Exp. 3). These results show that VOCs released by pre-infested chilli plants play an important role in mediating the attraction of female *B. tabaci*.

### Free-choice bioassay

**Figure 1:**
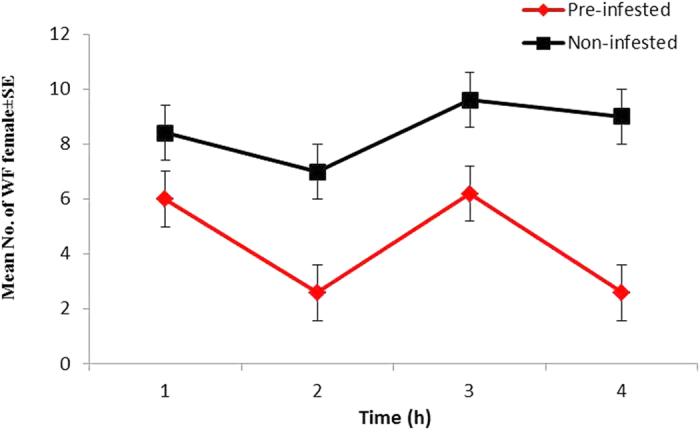
Mean (±SE) numbers of *B. tabaci* females settled on non-infested and pre-infested chilli plants at different times (1, 2, 3, 4 h) after WF release under free-choice bioassay, using two-way ANOVA.

**Figure 2:**
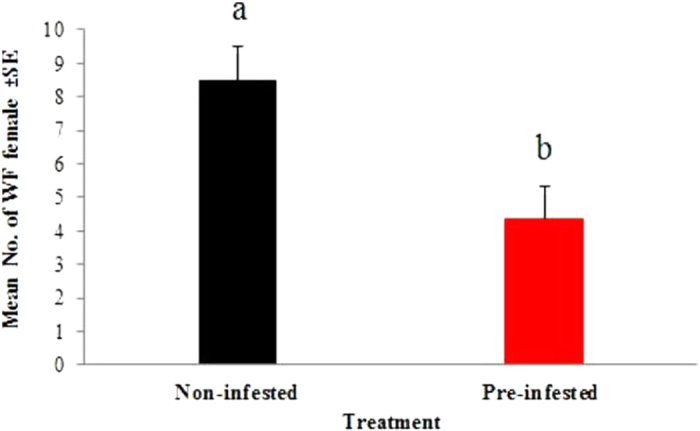
Total mean (±SE) number of settled *B. tabaci* females in free-choice bioassay with non-infested and pre-infested chilli plants. Different letters indicate a significant difference at (*P* < 0. 05). Each bar represents the mean of 5 replicates each assessed at 4 time intervals, using two-way ANOVA.

There was a significant difference in the number of WFs settling between treatment plants, but not between the times of response to host plants (Table 2 and Figure 1). No significant interaction between treatment and time was found (Table 2). Significantly more WFs were found on non-infested than on pre-infested chilli plants regardless of time after release (Figure 2).

### Volatile organic chemicals released by pre-infested and non-infested chilli plants

In total, 34 compounds were detected from pre-infested and non-infested chili plants, including terpenoids (monoterpenes, triterpenes, and sesquiterpenes), ketones, aldehydes, esters, hydrocarbons, fatty acids, and esters (Table 3). We confirmed that differences exist in volatile emissions of plants pre-infested with aphids compared to non-infested plants. The monoterpene emissions from pre-infested plants were significantly higher than non-infested plants in the following compounds: cymene (*t* = −5.66, df = 4, P = 0.005); and 1,8-cineole (*t* = 4.71, df = 4, P = 0.042). Emissions of some sesquiterpenes were also significantly higher in pre-infested, than non-infested, plants, including α-copaene (*t* = 4.50, df = 4, P = 0.045) and β-cadinene (*t* = 55, df = 4, P < 0.000). In addition, pre-infested plants released one ester compound (methyl salicylate) that was absent from the headspace of non-infested plants. In contrast, two different volatile compounds, eicosane and α-humulene, were released at higher levels from headspace samples of non-infested plants compared to pre-infested plants. On the other hand, no clear quantitative differences were observed between plant treatments in aldehydes, fatty acids, ketones, triterpenes and hydrocarbons (Table 3).

## Discussion

Our results demonstrate that aphid infestation influenced the release of volatile compounds from chilli plants, which in turn influenced the behavioural response of female WF to host plants. The strongest evidence was provided by the olfactometer experiments, in which the WFs had no visual or physical contact with the plants and significantly preferred the odour of non-infested chilli plants (Table 1). This preference was also reflected in the results of free-choice bioassay, where WFs chose non-infested plants more often than pre-infested plants (Figure 2).

The ability of female *B. tabaci* to discriminate between non-infested chilli plants and those pre-infested by aphids suggests that the response of WF to aphid-infested plants was affected by volatile compounds released by the plants. During probing of the leaves aphids puncture virtually all mesophyll cells on their path to a major vein of the phloem^19^. Salivary proteins injected by aphids while feeding on plants are known to be directly involved in triggering plant responses to insect herbivores^20^,^21^. Guerrieri *et al*. (1993)^22^ found that aphid infestation appeared to induce volatile emissions that repelled further infestation by other aphids. Similarly, resistance induced by the spider mite *Tetranychus turkestani* feeding on cotton seedlings reduced WF densities^23^.

The differences in preference of female WFs to pre-infested and non-infested chilli plants (Table 2) could be attributed to qualitative and quantitative differences in volatile compounds emitted from the plants. Some of these compounds may act as direct^24^ or indirect defences against specific herbivores^25^, and may influence the host-plant selection process of other herbivores^26^. For example, the large quantities of methyl salicylate (MeSA) that were detected in pre-infested chilli plants may have been induced by aphid infestation, as reported in other plant species such as lima bean, *Arabidopsis thaliana*, tomato, alfalfa, and soybean^27^,^28^,^29^,^30^,^31^,^32^. MeSA has been reported to be involved in plant defence, particularly in the elicitation of systemic acquired resistance (SAR)^33^. Zhu and Park (2005)^31^ and Pareja *et al*. (2009)^32^ identified MeSA as a good indicator of aphid feeding on soybean and alfalfa plants. Girling *et al*. (2008) also reported that aphid infestation induced release of MeSA in *Arabidopsis thaliana* (Brassicales: Brassicaceae). In another study, MeSA showed antifeedant activity against pine weevils^34^. Moreover, a positive electroantennogram response was shown when MeSA was applied to the antennae of *Coccinella septempunctata* (Coleoptera; Coccinellidae), and this predator and syrphid flies were attracted to MeSA-baited traps^31^. Our study also confirmed that aphid-infested chilli plants exhibited changes in the level of MeSA release that could be responsible for the effective resistance response of chilli plants against WFs; it may thus be advantageous for WFs to avoid chilli plants that produce this compound.

In general, plants infested with aphids showed altered terpene release profiles^35^,^36^, including induced release of α-pinene, β-pinene, cymene, α-phellandrene and d-limonene^37^. In our study, aphid infested chilli plants were shown to have increased release of volatile monoterpenes compared to non-infested plants (Table 3). Production of the monoterpenes cymene and 1,8-cineole was significantly increased in pre-infested chilli plants compared with non-infested plants. These compounds are known to be repellent to insects^38^. Recently, Yang *et al*. (2010)^39^ noted that ginger oil extract repelled adult WFs in a vertical olfactometer experiment. Repellent properties of this essential oil appear to be associated with a mixture of constituents including monoterpenes (1,8-cineole, phellandrene, camphene, α-pinene, myrcene, citral, and borneol), sesquiterpenes, aldehydes, and alcohols^40^,^41^,^42^,^43^. Similarly, *B. tabaci* has been reported to prefer cultivated tomato varieties over wild tomatoes, which was attributed to high levels of the monoterpenes *p*-cymene, *g*-terpinene, and *b*-myrcene being released by wild tomato plants^44^,^45^. 1,8-Cineole also showed significant antifeedant activity against *Tribolium castaneum*^46^. It is possible that WFs could respond to honeydew or other aphid-associated cues on pre-infested plants, however, bioassay conditions did not lead to noticeable honeydew production nor microbial growth. To our knowledge, there are no studies that report the volatile profiles of honeydew produced by *M. persicae*. However, twelve volatiles were found in honeydew produced by *Megoura viciae*, most of which were fermentation-associated products with a butane core^47^. None of these compounds, with the exception of limonene, was found in the volatile profile of pre-infested plants in our study. Limonene was found in both non-infested and pre-infested plant volatiles, but there was no significant difference in limonene production among plant types. Therefore, honeydew and associated aphid cues do not appear to have influenced WF behavior. These results support the conclusion that aphid infestation plays a role in volatile compound-induced resistance to WF infestation in chilli.

This study provides new evidence that infestation by aphids affects the defences of chilli plants against WFs by inducing the emission of various volatile compounds, which subsequently have an adverse (repellent) effect on *B. tabaci* females. Further study is needed to evaluate the selected VOCs for their behavioral effects on *B. tabaci* in order to determine which compounds have the most adverse effect on host plant selection. The data from such studies could enhance the IPM program for WFs by using commercially produced VOCs as repellents or attractant traps for WFs in the field.

## Methods

### Host plants

Chilli (*Capsicum annuum* var. Kulai) seeds were obtained from the Malaysian Agriculture Research & Development Institute (MARDI) Station, Jalan Kebun, Klang. Seeds were placed in distilled water for 8 days to germinate, after which they were placed in hydroponic solution (100.30 kg Ca (NO_3_)_2_, 790 g iron chelate, 2.63 kg K_2_HPO_4_, 5.83 kg KNO_3_, 5.13 kg MgSO_4_, 30 g H_3_BO_3_, 61 g MnSO_4_, 3.9 g CuSO_4_, 3.7 g (NH_4_)_2_MoO_4_, and 4.4 g ZnSO_4_ dissolved in 100 L water) in plant cups. The plant cups were placed into holes cut in a cylindrical piece of polystyrene through which the hydroponic solution flowed. The plants selected for the experiments were at least 30 d old and were at the nine- or ten-leaf stage.

### Insect rearing

Green peach aphids, *Myzus persicae* (Sulzer), were established from apterous adult aphids collected from *C. annuum* plants grown in a glasshouse at MARDI. *Myzus persicae* individuals were reared and maintained on *C. annuum* plants in a growth chamber under controlled laboratory conditions (20 ± 2 °C; 60–70% relative humidity [RH]). The aphids were provided with new chilli plants weekly. *Bemisia tabaci* were collected in the field at MARDI and reared on *C. annuum* in insect-proof mesh cages (60 × 60 × 60 cm) in a greenhouse at 30–36 °C and at 50–60% RH. Newly emerged female WFs were collected and starved for 2 h before the beginning of each trial.

### Pre-infestation of host plants

Four-week-old chilli seedlings were covered with plastic tubes (15 cm dia, 30 cm high) and a total 100 aphids per plant were carefully released into the tube with a fine brush. Chilli plants infested with adult aphids were held for 3 days in a growth chamber with environmental conditions of 20 ± 2 °C and a photoperiod of 16 h L: 8 h D. The aphids were then removed carefully with a paintbrush before the experiments. Non-infested plants were used as the control treatment, and were maintained under the same conditions but were not exposed to aphids.

### Olfactory choice experiment

**Figure 3:**
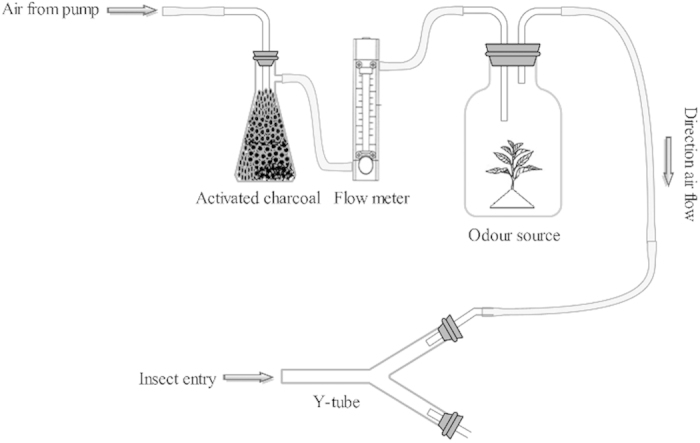
A schematic diagram of the Y-tube olfactometer, modified from Akol *et al*. (2003)^48^, used to test the effect of *M. persicae* feeding on behavioural response of *B. tabaci*.

Olfactory-choice bioassays to assess *B. tabaci* responses to volatile organic compounds (VOCs) produced by chilli plants pre-infested with *M. persicae* were conducted using a Y-tube olfactometer, as previously described by Akol *et al*. (2003)^48^ (Figure 3) with some minor modifications in the size of Y-tube (0.8 cm i.d., 10-cm-long base, two 10-cm branches at a 45° angle from one another). Plants were placed inside two 3-L glass containers, one affixed to each arm of the olfactometer with silicone tubes. The VOCs were circulated through the system using pressure pumps (Cole-Parmer Air cadet vacuum/pressure station, Vernon Hills, Illinois, USA). Air was pumped through an active charcoal filter prior to passing through two flow meters, which channelled the air at 60 mL/min into the two glass containers, where it passed through the odour source, and then into the two arms of the olfactometer. Female WFs were exposed to all of the potential stimulus treatment pairs (Exp. 1: pre-infested with aphids vs. non-infested; Exp. 2: pre-infested with aphids vs. clean air; Exp. 3: non-infested vs. clean air). Individuals were released within the first centimetre of the olfactometer base tube and their responses were measured for 10 min. Insects that walked at least 4 cm into one of the arms and did not return after 15 s were considered to have made a final choice. Insects that did not make a decision within the 10-min limit were excluded from the results. Each experiment was repeated five times for each combination of stimulus pairs, and each replicate consisted of 10 adult female WF assayed individually (i.e. total n = 50 for each treatment). The assays were conducted at 24 °C and 65–75% RH. Assay equipment was washed using soap and water and thoroughly sterilized with cotton wool drenched in 70% ethanol between each replicate to avoid the possibility of contamination by odours left from previous replicates.

### Free-choice bioassay

To investigate the preference of *B. tabaci* between plants pre-infested with aphids and non-infested plants in the presence of both olfactory and visual cues, release–recapture experiments were performed. Alternating non-infested and pre-infested chilli plants were arranged in a circle inside wooden cages covered with insect-proof nets (60 × 60 × 60 cm), equidistant from a central insect release point. There were 5 replicate cages, and each cage contained 10 plants (5 non-infested and 5 pre-infested with aphids). Light was provided by high-pressure sodium lamps and cages were maintained in laboratory conditions at 24 °C and 65% RH. Female whiteflies (n = 300 per cage) were starved for 2 h prior to release from glass vials at the centre of each cage. The numbers of whiteflies settled on the underside of 3 leaves per plant were counted at 1, 2, 3, and 4 h after release. One sample leaf was selected from each of three strata (upper, middle, lower) on the plant.

### Collection and analysis of VOCs

**Figure 4:**
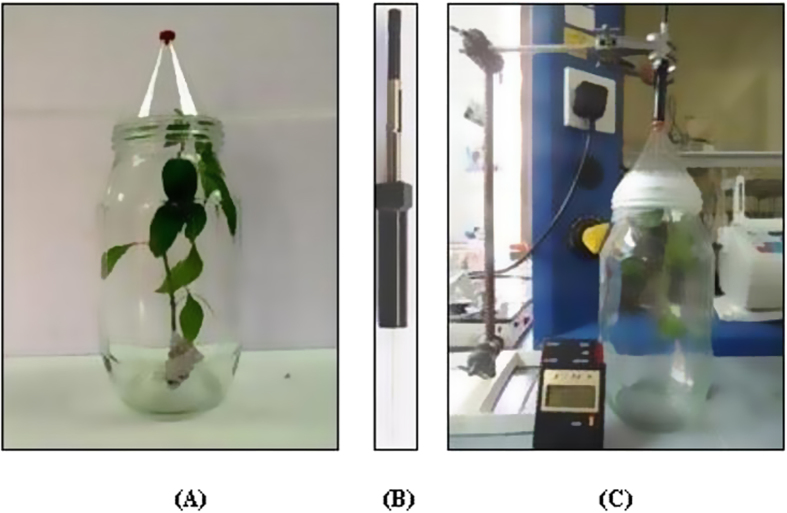
Static headspace sampling with (SPME) device. (**A**) The plant sample was enclosed in a glass container for 60 min, with a broad opening for easy removal of the plant. (**B**) The fiber is mounted on a SPME fiber holder. (**C**) The fiber was injected through the septum of the sample container by pushing the plunger of the SPME fiber holder, and extended from the needle and exposed to volatiles for 30 min. After collection VOCs, the fiber was retracted into the needle and the SPME device removed from the container for GC-MS analysis.

The VOCs emitted by pre-infested and non-infested chilli plants were collected using a static-headspace sampling device with a solid-phase microextraction (SPME) fibre coated with polydimethylsiloxane/divinylbenzene (PDMS/DVB, 65 μm). Each plant sample with 9 to 10 leaves was enclosed in a 3 L glass container for 60 min, and the SPME fibre was extended into the headspace to collect volatiles for a fixed 30 min time period. The glass chambers contained large openings for easy insertion and removal of plant sample (Figure 4). After collection of volatile substances at (23 ± 1 °C and 60% ± 5% RH), the SPME fibre was inserted directly into a thermal desorption gas chromatograph-mass spectrometer (Shimadzu, GC-MS QP-2010 model), with a DB5-MS column (30 m × 25 mm × 0.25 μm film thickness). The fibre was left in the injector (on splitless mode) for 2 min at a final temperature of 250 °C (initial temperature of 40 °C for 5 min hold, and increased by 3 °C/min until reaching 250 °C). Helium (1 mL/min) was used as the GC carrier gas. The identification of separated compounds was conducted using a NIST 2008 spectral library, matching retention time and mass spectra with those of authentic standards. The relative quantities of each volatile compound were estimated based on its peak area shown by mass spectrometry. Three non-infested and three pre-infested chili plants were used in the analyses.

### Statistical analyses

Paired *t*-tests were used to compare the behavioural responses of female WFs to odour source pairs in the olfactometer assays. Free-choice bioassay data were analysed by two-way ANOVA, where treatment and time were independent variables, and number of female WF responding was the dependent variable. Differences in the total peak area of each VOC produced by pre-infested and non-infested chilli plants were analysed using unpaired two sample *t* -tests with the exception of one-tailed *t*- testing for zeros variances, at *P* < 0.05. All statistical analyses were conducted using the Minitab Statistical Package (Version 16).

## Additional Information

**How to cite this article**: Saad, K. A. *et al*. Aphid-induced Defences in Chilli Affect Preferences of the Whitefly, *Bemisia tabaci* (Hemiptera: Aleyrodidae). *Sci. Rep*. **5**, 13697; doi: 10.1038/srep13697 (2015).

## Figures and Tables

**Table 1:** Olfactory response of *B. tabaci* females in olfactometer experiments given a choice between VOCs from chilli plants: (1) pre-infested by aphids and non-infested; (2) pre-infested and clean air; and, (3) non-infested and clean air.

Experiment	Proportion (±SE) of WF females responding		*P*-value
1	Pre-infested	Non-infested	0.041
	0.352 ± 0.063b	0.647 ± 0.63a	
2	Pre-infested	Clean Air	0.011
	0.332 ± 0.037b	0.667 ± 0.037a	
3	Non-infested	Clean Air	0.58
	0.610 ± 0.055a	0.389 ± 0.055a	

Means (±SE) with different letters in each row significantly differ, using *t*-tests (for dependent-samples) (*P* < 0.05).

**Table 2:** Results of two-way ANOVA for mean numbers of *B. tabaci* settling on treatment plants at different times after release in free-choice bioassay.

Source	df	Sum of squares	F-value	*P*-value
Time	3	19.35	0.87	*P* > 0.05
Plants (Treatment)	1	172.22	7.72	*P* < 0.05
Time × plants	3	7.292	0.33	*P* > 0.05
Error	39	22.30		

**Table 3:** Quantities of major volatile compounds released by non-infested and aphid pre-infested chilli plants through headspace sampling by SPME.

Volatile compound	RT	Non-infested	Pre-infestation by aphids	*P*-value
Monoterpenes				
α-Pinene	7.73	0.060 ± 0.045	0.006 ± 0.006	0.386
β-Pinene	9.23	0.023 ± 0.023	0.006 ± 0.003	0.525
Limonene	10.85	0.410 ± 0.22	0.217 ± 0.068	0.173
p-Cymene	10.6	0.016 ± 0.008	0.043 ± 0.003	**0.005**
Geranylacetone	23.23	0.323 ± 0.117	0.137 ± 0.078	0.155
β-2-Carene	11.65	0.076 ± 0.076	0.010 ± 0.005	0.250
y-Terpineol	16.21	0.00	0.130 ± 0.045	0.051
α-Terpineol	16.13	0.00	1.11 ± 1.11	0.211
Terpinen-4-ol	15.63	0.00	0.050 ± 0.050	0.211
Camphor	14.71	0.00	0.403 ± 0.143	0.053
1,4-Cineole	9.96	0.00	0.050 ± 0.050	0.374
1,8-Cineole	10.7	0.00	0.056 ± 0.012	**0.021**
Borneol	15.45	0.00	0.140 ± 0.049	0.052
Triterpenes				
Squalene	56.18	1.690 ± 0.88	0.263 ± 0.206	0.251
Sesquiterpenes				
α-Humulene	23.55	0.723 ± 0.078	0.00	**0.012**
(E)-Caryophyllene	22.73	0.82 ± 0.79	1.44 ± 1.20	0.459
Copaene	21.26	0.157 ± 0.123	0.010 ± 0.006	0.360
β -Cadinene	25.08	0.00	0.366 ± 0.006	**0.000**
α-Copaene	21.21	0.00	0.143 ± 0.044	**0.045**
Aldehydes				
Nonanal	13.16	0.243 ± 0.158	0.217 ± 0.217	0.792
Decanal	11.96	0.433 ± 0.069	0.357 ± 0.251	0.716
Octanal	10.01	0.00	0.143 ± 0.143	0.423
Ketones				
5-Hepten-2-one,6-methyl	9.41	0.070 ± 0.032	0.00	0.161
Fatty acids				
Hexadecanoic acid	35.16	0.237 ± 0.109	0.00	0.645
Hydrocarbons				
Eicosane	37.71	0.513 ± 0.062	0.073 ± 0.038	**0.009**
Tetradecane	21.7	0.197 ± 0.068	0.653 ± 0.470	0.452
Hexacosane	48.45	4.99 ± 2.0	2.62 ± 2.6	0.081
Tridecane	21.56	0.286 ± 0.072	0.1000 ± 0.005	0.123
Undecane	12.76	0.297 ± 0.061	0.290 ± 0.051	0.954
Dodecane	15.85	0.297 ± 0.073	0.170 ± 0.085	0.331
Heptacosane	45.58	3.04 ± 1.03	1.56 ± 1.56	0.605
Pentadecane	24.35	0.086 ± 0.052	0.033 ± 0.033	0.582
Decane	9.3	0.250 ± 0.056	0.296 ± 0.066	0.663
Ester				
Methyl salicylate	16.06	0.00	0.056 ± 0.012	**0.042**

RT = retention time.

Each value represents the mean peak areas (±SE) of 3 replicates. *P* values in boldface indicate significant differences between the means for non-infested and pre-infested plants in that row, at α = 0.05, using *t*-tests (one-tailed; two-tailed).
